# Gambling Venues and Their Association With Incidence of Help-Seeking Gambling Disorder

**DOI:** 10.1192/bjo.2025.10488

**Published:** 2025-06-20

**Authors:** Konstantinos Ioannidis, Dianna Smith, Samuel R. Chamberlain

**Affiliations:** 1 University of Southampton, Southampton, United Kingdom; 2 Hampshire and Isle of Wight Healthcare NHS Foundation Trust, Southampton, United Kingdom

## Abstract

**Aims:** The NHS Southern Gambling Service (SGS) opened in 2022, and provides evidence-based assessment and treatment for people affected by gambling disorder across the South East of England. It is known that gambling venues are often placed in highly deprived areas, where populations vulnerable to gambling disorder reside. Little is known about whether geographical presence of gambling venues is linked to higher rates of referrals for gambling disorder to clinical services. The aims were to draw insights on the association between the incidence of referrals at the SGS and number of registered gambling venues across the geographical footprint of the regional service, while we adjust for indices of multiple deprivation.

**Methods:** Service level data for referrals per Low-Tier Local Authority (LTLA) level were merged with open access national datasets for indices of multiple deprivation (Office for National Statistics 2021) and number of gambling venues in each area (Gambling Commission 2024). Linear regression analyses were performed in-sample to identify the strength of the associations between the number of referrals and number of registered venues, adjusted for indices of multiple deprivation (IMD). This service evaluation was pre-registered with the Hampshire and Isle of Wight Healthcare NHS Foundation Trust Clinical Effectiveness team. All data analysis was conducted in R version 4.4.2.

**Results:** A total of 668 participants were referred to the SGS from September 2022 to end of November 2024. The correlation between venues and referral incidence was strong (Pearson’s r=0.58, p<0.001). Number of venues per LTLA were statistically associated with incidence of referrals to the SGS (t=3.9, p<0.001) including after adjusting for IMD indices. The model which included only the number of venues as a predictor explained 33.3% of the variance in incidence rate (R²=0.3325, p<0.001).

**Conclusion:** Number of gambling venues was strongly associated with incidence of referrals to the SGS. This association remained strong even after adjusting for indices of multiple deprivation. These insights can help the SGS in the strategic planning of development and utilization of its future resources, and highlight the need to examine sources of referrals nationally and links to contextual factors such as presence of gambling venues. Further work is warranted to define the optimal granularity for dissecting the geospatial links between the location of gambling venues and referrals to NHS Gambling Treatment Services, to further establish the stability and generalizability of these findings, as well as to explore a broader range of implicated bio-socio-economic factors.

